# Making the invisible visible: searching for human T-cell lymphotropic virus types 1 and 2 (HTLV-1 and HTLV-2) in Brazilian patients with viral hepatitis B and C

**DOI:** 10.1590/0074-02760170307

**Published:** 2018-02

**Authors:** Adele Caterino-de-Araujo, Fabiana Aparecida Alves, Karoline Rodrigues Campos, Marcílio Figueiredo Lemos, Regina Célia Moreira

**Affiliations:** 1 Secretaria de Estado da Saúde de São Paulo, Coordenadoria de Controle de Doenças, Instituto Adolfo Lutz, Centro de Imunologia, Laboratório de Pesquisa em HTLV, São Paulo, SP, Brasil; 2 Secretaria de Estado da Saúde de São Paulo, Coordenadoria de Controle de Doenças, Instituto Adolfo Lutz, Centro de Virologia, Núcleo de Doenças de Transmissão Sanguínea e Sexual, Laboratório de Hepatites, São Paulo, SP, Brasil

**Keywords:** HBV, HCV, HIV, HTLV-1/-2, co-infection

## Abstract

With this study, the authors hope to alert clinicians regarding the presence of human T-cell lymphotropic virus type 1 and 2 (HTLV-1/-2) infections in patients with viral hepatitis B and C in Brazil. HTLV-1/-2 were detected in 1.3% of hepatitis B virus (HBV)- and 5.3% of hepatitis C virus (HCV)-infected blood samples sent for laboratory viral load measurements. A partial association of human immunodeficiency virus (HIV)-1 and HTLV-1/-2 infection was detected in patients with HCV (HIV+, 27.3%), whereas this association was almost 100% in HBV-infected patients (HIV+, all except one). The high prevalence of HTLV-1/-2 infection among patients with hepatitis C was of concern, as HTLV-1/-2 could change the natural course of subsequent liver disease. The authors suggest including HTLV-1/-2 serology in the battery of tests used when following patients with viral hepatitis in Brazil, regardless of the HIV status.

Human retroviruses, particularly human immunodeficiency virus (HIV)-1 and the human T-cell lymphotropic virus types 1 and 2 (HTLV-1 and HTLV-2), are endemic in Brazil (Catalan-Soares et al. 2005, Gessain & Cassar 2012, MS 2016a). Notably, Brazil has gained international notoriety in the fight against acquired immunodeficiency syndrome (AIDS) by providing free and universal access to antiretroviral treatment for all patients and promoting educational programs aimed at blocking virus transmission/acquisition. Although HTLV-1 is the cause of at least two diseases with high mortality and morbidity, adult T cell leukaemia/lymphoma (ATLL) and HTLV-1-associated myelopathy/tropical spastic paraparesis (HAM/TSP) (Gessain & Cassar 2012), HTLV-1/-2 are not considered infections requiring obligatory notification in Brazil. In contrast, viral hepatitis are considered an important global health problem, since they may progress to chronic liver diseases. Such occurrences are the major cause of liver transplantation among patients infected with the hepatitis C virus (HCV) in developed countries. In Brazil, patients infected with viral hepatitis are subject to compulsory notification, and hepatitis B virus (HBV) and HCV infections have received significant attention from the Ministry of Health (MH).

The Epidemiological Bulletins of HIV/AIDS and Viral Hepatitis from the Brazilian MH disclose the number of infected individuals according to geographic region, state, sex, and age (MS 2016a, b). However, regional socio-demographic characteristics have led to the underestimation of some data (MS 2016b). Interestingly, the Southeast region of Brazil, which is the most populous and of the highest incomes in the country, accounts for the majority of cases of HIV-1, HBV, and HCV infection reported to the Brazilian MH (MS 2016a, b) (Fig. 1).

**Fig. 1:**
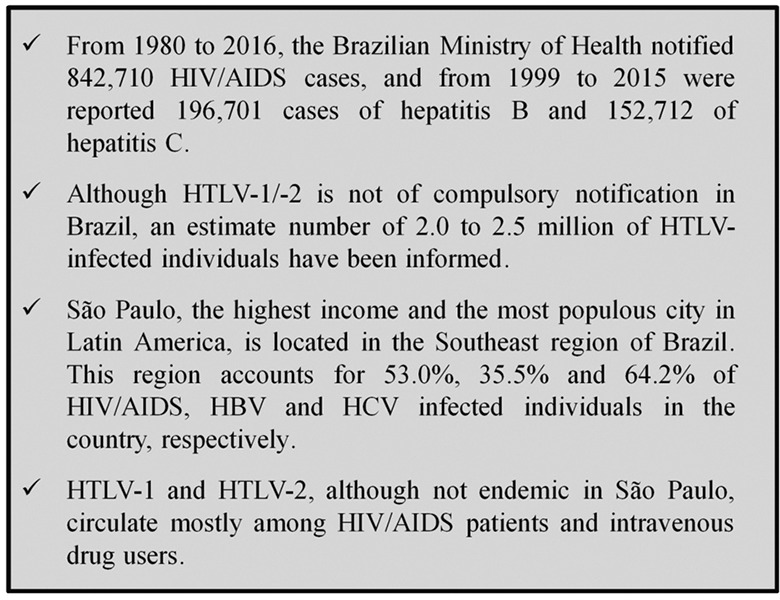
estimated numbers of individuals infected with HIV-1/AIDS, HTLV-1/-2, HBV and HCV in Brazil.

In Brazil, HTLV-1 is endemic among individuals of African origin (Dourado et al. 2003, Paiva & Casseb 2015), whereas HTLV-2 is endemic among the native populations from Amazonia, as well as intravenous drug users (IDU), regardless of the HIV/AIDS status (Alcântara Jr et al. 2003, Ishak et al. 2003, Caterino-de-Araujo et al. 2015, Paiva & Casseb 2015). The Guideline for the Clinical Management of HTLV Infection is a unique document produced by the Brazilian MH regarding HTLV (MS 2013). However, the Clinical Protocol and Therapeutic Guidelines for HIV in Adults, also published by the Brazilian MH, recommend that an HTLV serologic evaluation be performed at least once during the follow-up of HIV-1-infected individuals (MS 2014). Specific serological testing is recommended, as HTLV-1 infection could cause an increase in the CD4+ cell count, which is used to evaluate HIV-1 infection control (Brites et al. 2009, MS 2014).

Although HIV-1, HTLV-1/-2, HBV, and HCV share some common routes of infection, the risks of acquiring these infectious agents vary considerably in Brazil, and are dependent on the genetic backgrounds of individuals, geographic region, socioeconomic conditions, and risk behaviours (Catalan-Soares et al. 2005, Pereira et al. 2013, Ximenes et al. 2015, MS 2016a, b, Assone et al. 2016).

Dual and/or triple co-infections involving HIV-1, HTLV-1, HTLV-2, HBV, or HCV can occur and may contribute to changes in the natural course of each subsequent viral disease. For instance, HIV/HTLV-1 co-infection has been associated with a rapid progression to AIDS and a shorter survival time (Brites et al. 2001). Furthermore, the HTLV-1-associated increase in CD4+ cell counts may not provide an immune benefit to patients (Brites et al. 2009). In contrast, HIV/HTLV-2 co-infection has been associated with a slower progression to AIDS and longer survival time (Casoli et al. 2000, Beilke 2012). HCV/HTLV-1 co-infected patients harbour less severe hepatic injuries, as confirmed by lower levels of serum liver aminotransferases, other liver enzymes, and fibrosis, when compared with their HCV and HCV/HIV co-infected counterparts (Cardoso et al. 2009, Bahia et al. 2011, Espíndola et al. 2017). In addition, studies on HTLV-1 and HIV/HTLV-1 co-infected patients from Brazil found the spontaneous clearance of HCV and/or lower rate of HCV viremia detection (Moreira et al. 2013, Le Marchand et al. 2015). In contrast, a study in the USA described an increase in the HCV viral load among HIV and/or HTLV-2 co-infected individuals (Hisada et al. 2003). Regarding HBV infection, HIV-1 and HTLV-1 single-infected individuals were found to have a higher rate of HBV antigenemia, suggesting reduced HBV clearance, and HIV/HTLV-1 co-infected patients showed no protective effects of these retroviruses against hepatitis B (Moreira et al. 2013, Marr et al. 2017). Taking together, HTLV-1 seems to positively affect HCV clearance and negatively affect HBV removal, whereas HTLV-2 has a negative effect on HBV clearance. Consequently, co-infections with HTLV-1 and HTLV-2 exert different effects in patients with viral hepatitis, as well as in those with HIV-1 infection.

The mechanism by which viral co-infections might affect the outcomes of subsequent diseases is not completely understood, although some hypotheses have been devised regarding the higher odds of HCV clearance in HTLV-1 and/or HIV/HTLV-1 co-infected patients. Changes in the immunological environment, particularly the balance between the Th2 immunological response induced by HCV and the Th1 response induced by HTLV-1, could be an explanation. Specifically, an increase in the spontaneous production of IL-1, IL-2, and IFN-g, reduction in the production of IL-4 by mononuclear cells, and elevated serum levels of pro-inflammatory cytokines might explain this finding (Le Marchand et al. 2015, Silva et al. 2016, Espíndola et al. 2017).

However, as the benefit of HTLV-1 co-infection had not been confirmed in patients with hepatitis C from other parts of the world, except in Brazil (Castro & Roger 2016), further studies were needed to confirm and clarify these issues.

Studies describing the clinical outcomes of HBV and HCV single-infected individuals versus those co-infected with HTLV-1/-2 in Brazil are scarce, and no studies have discussed the prevalence of such co-infections. Therefore, we conducted a preliminary study on the prevalence of HTLV-1/-2 among HBV- and HCV-infected individuals.

Plasma samples from 1,244 individuals with viral hepatitis were sent to Instituto Adolfo Lutz, a public health laboratory in São Paulo, for HCV and HBV viral load analysis. The samples were screened for HTLV-1/-2 infection using an enzyme immunoassay (EIA, HTLV-I/II, Gold ELISA, REM Ind. Com. Ltda, São Paulo, SP., Brazil), and were confirmed by a line immunoassay (INNO-LIA HTLV-I/II, Fujirebio, Europe N.V., Belgium). HIV-1 infection was investigated using an immunochromatographic assay (Rapid Check HIV 1 + 2, Universidade Federal do Espírito Santo, ES, Brazil). All assays were performed according to the respective manufacturers’ instructions. Plasma samples were collected from 622 HCV-infected individuals (G1, 343 males and 279 females), and 622 HBV-infected individuals (G2, 327 males and 295 females). The study was approved by the Ethics Committee for Research of Instituto Adolfo Lutz (CTC #21I-2016), under the MH protocol number CAAE - 55837316.0.0000.0059.

During the HTLV-1/-2 screening of 1,244 plasma samples, 44 reacted positively; 25 confirmed HTLV-1 infection (G1, 20 and G2, 5), 16 samples confirmed HTLV-2 infection (G1, 13 and G2, 3). Two samples were indeterminate (both G1), and one was negative (G2). The HTLV-1/-2 serology results by group (G1 and G2) and the HIV results are presented in the Table. Briefly, the overall prevalence of HTLV among 622 HCV-infected individuals was 5.3% (3.2% HTLV-1 and 2.1% HTLV-2); the corresponding prevalence among 622 HBV-infected individuals was 1.3% (0.8% HTLV-1 and 0.5% HTLV-2). Of 33 HCV/HTLV co-infected patients, 27.3% were HIV positive, compared to 6.5% of their HCV-infected counterparts (p < 0.0001). Among HBV-infected patients, 5.6% were HIV positive, whereas seven of eight HBV/HTLV co-infected individuals were HIV positive. The median age did not differ between the HCV-infected and HCV/HTLV co-infected individuals (50.8 vs. 50.6 years old, p = 0.5678) or between HBV-infected and HBV/HTLV co-infected patients (45.7 vs. 53.5 years old, p = 0.1074). Notably, the majority of both HBV/HTLV and HCV/HTLV co-infected individuals were male (62.5% and 63.6%, respectively).

**TABLE:** Serological results of HTLV-1/-2 and HIV from 1,244 blood samples of patients infected with HCV (G1) and HBV (G2) in São Paulo, Brazil

Groups	Overall	HCV(G1)	HBV(G2)
HTLV-1/2*^a^*		n (%)	n (%)
		622	622
	HTLV-1	20 (3.2)	5 (0.8)
	HTLV-2	13 (2.1)	3 (0.5)
	Total	33 (5.3)	8 (1.3)
HIV*^b^*	HIV	40 (6.5)	35 (5.6)
	HIV/HTLV-1/-2	9 (1.4)	7 (1.1)
	HIV/HTLV-1	4 (0.6)	4 (0.6)
	HIV/HTLV-2	5 (0.8)	3 (0.5)
	Total	49 (7.9)	42 (6.7)

n: number of samples analysed; G1, HCV-infected group; G2, HBV-infected group.

a results obtained by enzyme immunoassay (Gold ELISA HTLV-I+II, REM, São Paulo, SP, Br), and confirmed by line immunoassay (INNO-LIA HTLV-I/II, Fujirebio, Europe N.V, Belgium);

b results obtained by immunochromatographic assay (Rapid Check HIV 1+2, UFES, ES, Br).

The results obtained from the present study indicate that 5.3% of HCV-infected individuals from the city of São Paulo were co-infected with HTLV-1/-2. This percentage is concerning, as it is higher than that reported in populations considered at “high risk” for HTLV infection, such as men who have sex with men (MSM) and HIV/AIDS patients. In fact, in one study on MSM from Campinas, a city 100 km distant from São Paulo, the HTLV-1/2 infection rate was 1.5% (Soares et al. 2014). In recent studies conducted in São Paulo, we detected prevalence rates of 3.1% (1.7% for HTLV-1, 1.3% for HTLV-2, and 0.1% for HTLV), 4.2% (2.1% for HTLV-1, 1.7% for HTLV-2, 0.1% for HTLV-1 + HTLV-2, and 0.3% for HTLV) among patients who attended different STD/AIDS Centres in São Paulo (Caterino-de-Araujo et al. 2015, Campos et al. 2017). In addition, one of those studies detected associations of HIV/HTLV-1/-2 infection with the female sex [odds ratio (OR), 3.26], black/pardo skin colour (OR, 2.21), HBV (OR, 4.27) or HCV infection (OR, 24.40), and IDU (OR, 30.01) (Caterino-de-Araujo et al. 2015). These data are interesting, as they demonstrate that both retroviruses circulate in São Paulo at similar rates and among people older than 40 years. Moreover, the association of HBV and HCV suggests that similar behavioural risks are involved in the acquisition of all these viruses.

Another important point that emerged from the present study was the finding that only 27.3% of HCV/HTLV-1-2 co-infected patients were also HIV-1 seropositive. As the Brazilian MH has recommended a HIV-1 serologic analysis during viral hepatitis follow-up and as HTLV-1/2 serology has been suggested during the follow-up of HIV-1-infected patients, we might speculate that HIV-1 seropositive patients could indirectly know their HTLV status. However, HIV-1 seronegative patients with HCV infection (72.7%) likely would not know their HTLV status.

In addition, although HCV/HTLV-1 co-infection has been associated with better outcomes among patients in Brazil (Cardoso et al. 2009, Bahia et al. 2011, Moreira et al. 2013, Le Marchand et al. 2015, Silva et al. 2016, Espíndola et al. 2017), co-infection with HCV/HTLV-2 was described as having the opposite effect in a study from the USA (Hisada et al. 2003). Thus, the screening and discrimination of HTLV-1 from HTLV-2 in patients with viral hepatitis has prognostic value.

Regarding hepatitis B, only 1.3% of patients were co-infected with HTLV-1/-2, and these data corroborate our previous finding of a minor OR for HBV/HTLV relative to HCV/HTLV co-infection among HIV/AIDS patients (Caterino-de-Araujo et al. 2015). Among HIV-1/HTLV co-infected individuals, IDU had an OR of 30.01 relative to individuals infected with HIV-1 alone. Therefore, we could speculate that IDU is the principal risk factor for acquiring HTLV among patients infected with HBV. In fact, all except one of the HBV/HTLV-1/-2 co-infected patients were HIV positive. In contrast, other routes of transmission/acquisition, such as blood transfusion or sexual intercourse, could account for the results obtained from HCV-infected patients. Overall, the HIV serology results detected in the present study are in accordance with those reported by the Brazilian MH throughout Brazil and in the Southeast region. In those regions, the reported HIV prevalence rates were 5.2% and 8.1%, respectively, among HBV-infected patients, and 10.0% and 9.6%, respectively, among HCV-infected individuals (MS 2016b).

Regarding patient sex, although HTLV-1/-2 acquisition tends to occur preferentially among female subjects (Gessain & Cassar 2012, Caterino-de-Araujo et al. 2015, Campos et al 2017), in the present study, male subjects with both HBV and HCV were more frequently co-infected with HTLV-1/-2, a finding that might be attributable to behavioural risk factors. Regarding patient age, the observation that older patients were infected with these viruses corresponded in part with the decades when IDU was one of the major routes of human retrovirus transmission in Brazil (Caterino-de-Araujo et al. 2015). Additionally, this situation correlated with the initial commercial availability of specific serological tests for viral diagnosis in Brazil. Screening for these viruses has since become mandatory in Brazilian blood banks, where HIV and HBV screening became obligatory in 1988 and HCV and HTLV-1/-2 in 1993.

Of note, the Global Virus Network Task Force on HTLV-1 recommends that transplant societies should make extensive use of screening assays and should instruct donors and recipients about HTLV-1/2 infection, transmission, and disease prevention, as high rates of ATLL and HAM have been detected in HTLV-1-infected patients after organ transplantation (Gallo et al. 2016).

Taken together, our data lead us to suggest the inclusion of HTLV serology in the battery of tests used to follow up hepatitis virus-infected patients, regardless of their HIV status, particularly in HTLV-1/-2-endemic regions of Brazil. Surveillance of the current statuses of these viral infections could assist physicians with performing accurate patient follow-ups (Fig. 2).

**Fig. 2:**
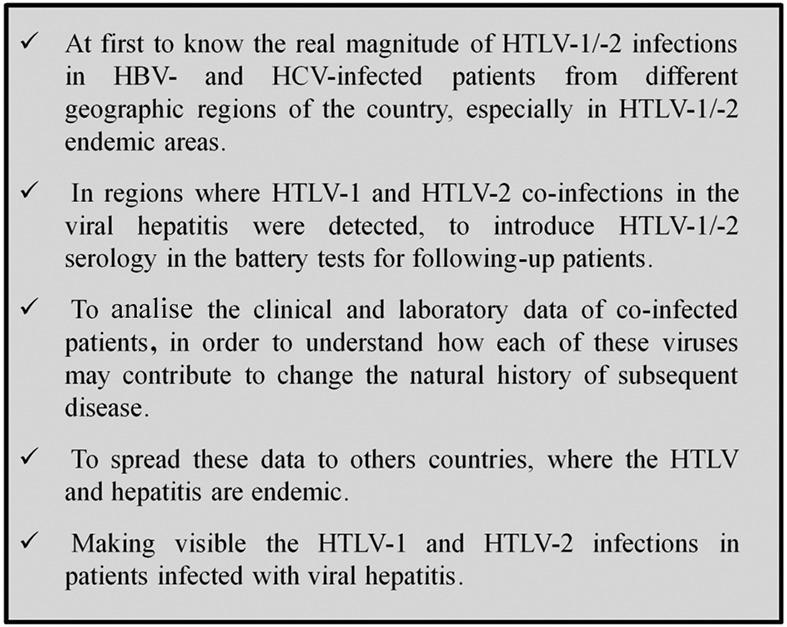
recommendation for following-up viral hepatitis B and C patients in Brazil.
